# Supplementary material for: Early-Phase Drive to the Precursor Pool: Chloroviruses Dive into the Deep End of Nucleotide Metabolism
Source: Viruses. 2023 Mar 31;15(4):911. doi: 10.3390/v15040911 (PMC10142491; doi:10.3390/v15040911)
Supplement: Supplementary file 1 [file viruses-15-00911-s001.zip › Table S4.pdf]

Table S4. *Chlorella variabilis* NC64A genome sequence information.

**T number**	T02924
**Org code**	cvr
**Aliases**	CHLVA, 554065
**Full name**	*Chlorella variabilis*
**Definition**	*Chlorella variabilis*
**Annotation**	KOALA
**Taxonomy**	TAX: 554065
**Lineage**	*Eukaryota*; *Viridiplantae*; *Chlorophyta*; *Trebouxiophyceae*; *Chlorellales*; *Chlorellaceae*; *Chlorella*
**Data source**	RefSeq (Assembly: GCF_000147415.1) BioProject: 223657
**Original DB**	JGI
**Keywords**	Unicellular photosynthetic green alga; intracellular photobiont; photosynthesis; adaptation to symbiosis
**Genome size**	46.2 Mb
**Statistics**	Number of protein genes: 9892, Number of RNA genes: 65
**Created**	2013
**Reference**	PMID: 20852019
**Authors**	[a]Blanc G, Duncan G, Agarkova I, Borodovsky M, Gurnon J, Kuo A, Lindquist E, Lucas S, Pangilinan J, Polle J, Salamov A, Terry A, Yamada T, Dunigan D, Grigoriev I, Claverie J-M, Van Etten JL
**Title**	[a]The *Chlorella variabilis* NC64A genome reveals adaptation to photosymbiosis, coevolution with viruses, and cryptic sex.
**Journal**	[a]Plant Cell 22:2943-55 (1) DOI: 10.1105/tpc.110.076406

[a]Blanc G, Duncan G, Agarkova I, Borodovsky M, Gurnon J, Kuo A, et al. The *Chlorella variabilis* NC64A genome reveals adaptation to photosymbiosis, coevolution with viruses, and cryptic sex. Plant Cell. 2010;22:2943-55 DOI: 10.1105/tpc.110.076406
