# Supplementary figures and images for: Early-Phase Drive to the Precursor Pool: Chloroviruses Dive into the Deep End of Nucleotide Metabolism
Source: Viruses. 2023 Mar 31;15(4):911. doi: 10.3390/v15040911 (PMC10142491; doi:10.3390/v15040911)

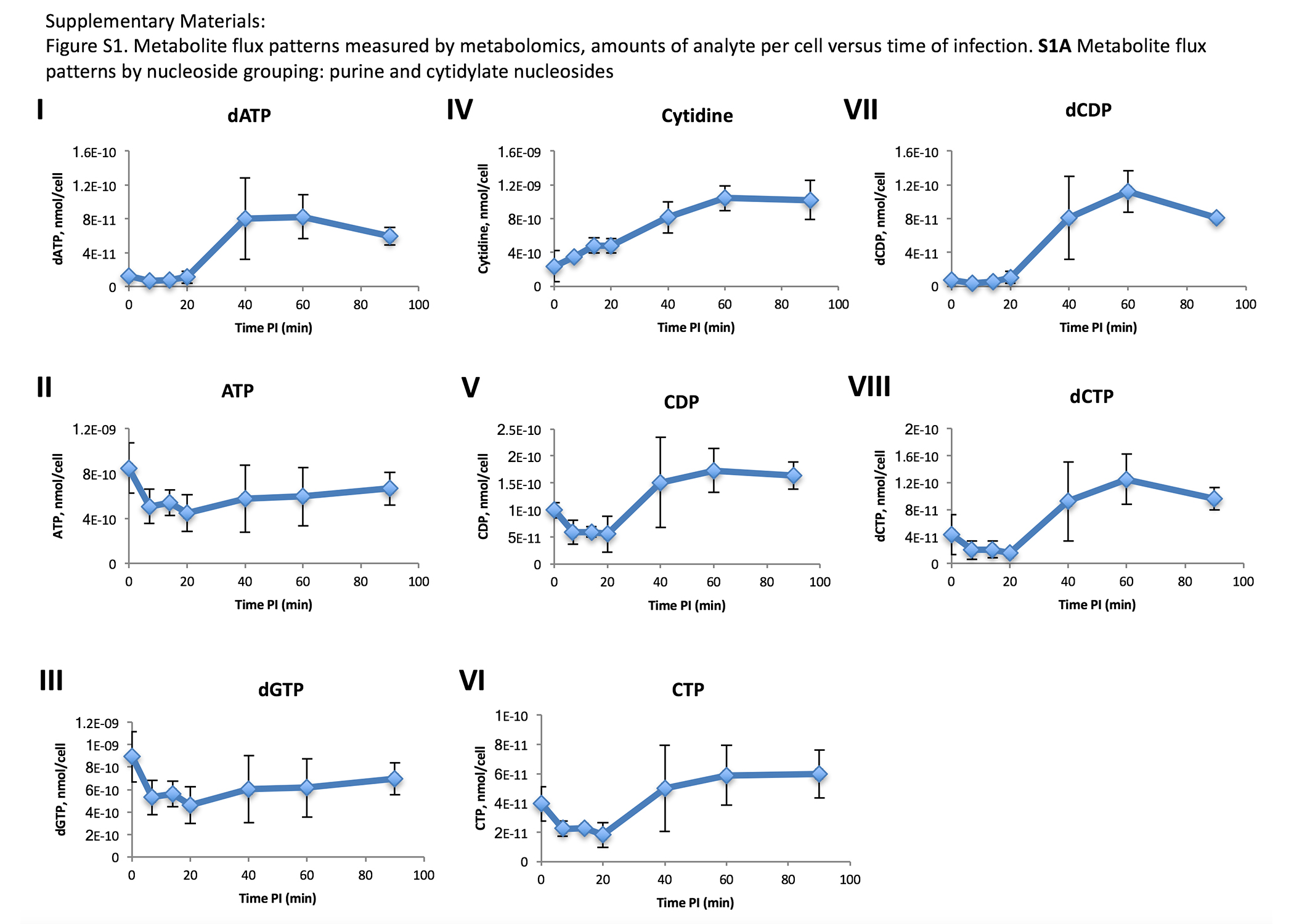

Supplement: Supplementary file 1 [file viruses-15-00911-s001.zip › Figure S1A.jpg]

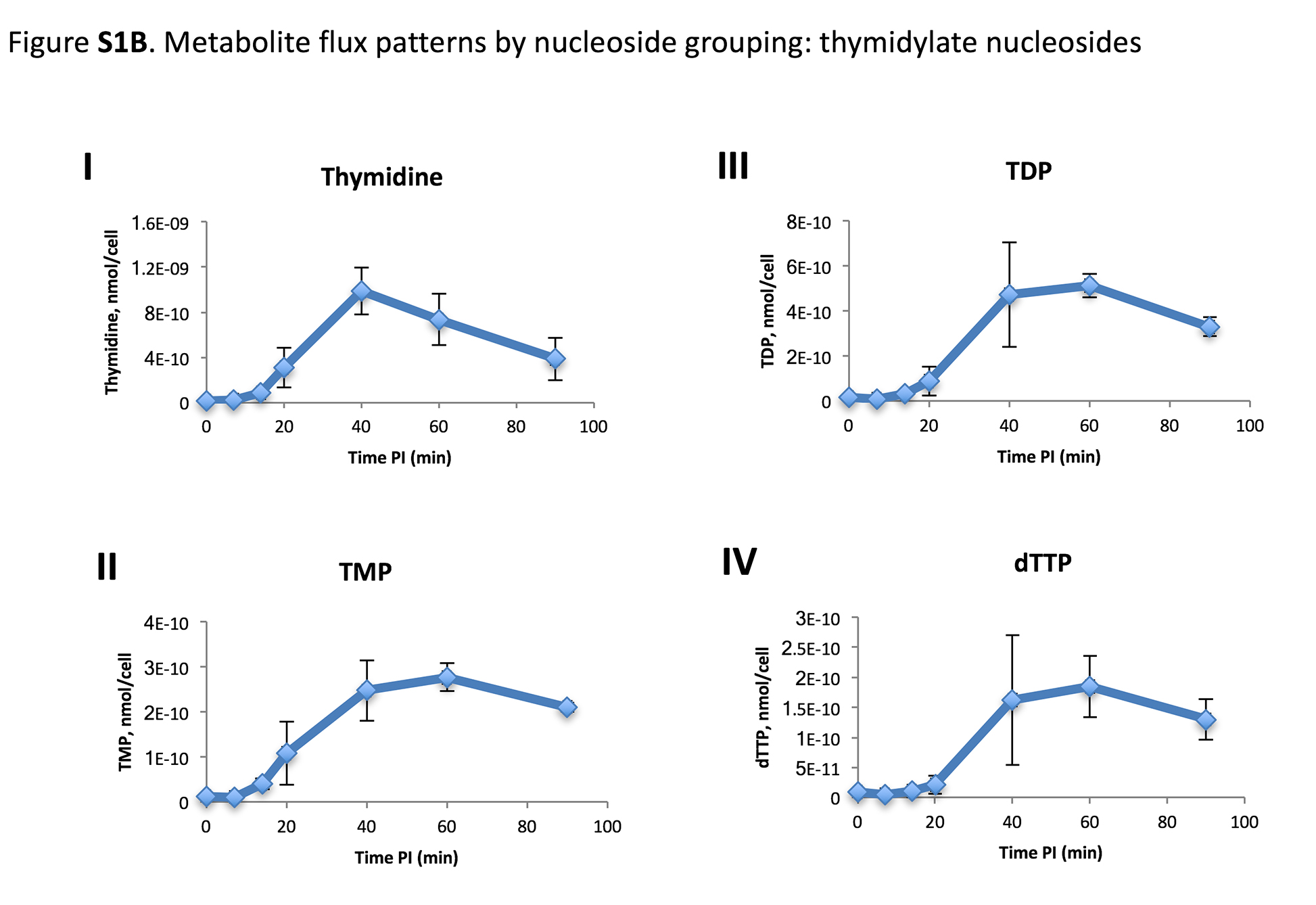

Supplement: Supplementary file 1 [file viruses-15-00911-s001.zip › Figure S1B.jpg]

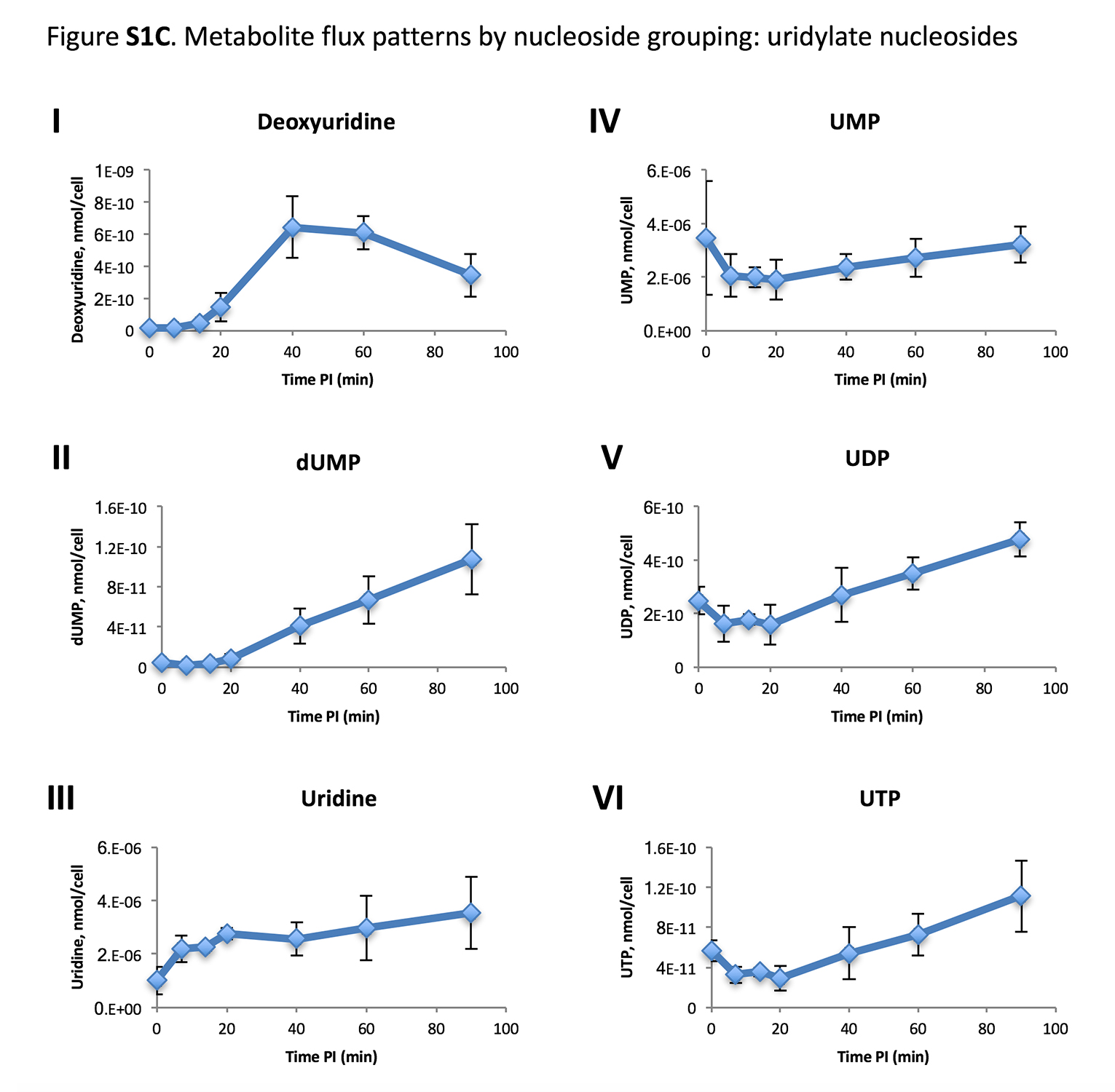

Supplement: Supplementary file 1 [file viruses-15-00911-s001.zip › Figure S1C.jpg]
